# The Analysis of *Solanum lycopersicum* Sap Dark Proteome Reveals Ordered and Disordered Protein Abundance

**DOI:** 10.3390/cimb47090769

**Published:** 2025-09-18

**Authors:** Francisco Antonio Reyes-Soria, Francisco Guillén-Chable, Enrique Castaño de la Serna, Lorenzo Felipe Sánchez-Teyer, Miguel Angel Herrera-Alamillo, Alejandro Pereira-Santana, Luis Carlos Rodriguez-Zapata

**Affiliations:** 1 Biotechnology Department, Yucatán Scientific Research Center A.C., Mérida CP 97205, Yucatán, Mexico; francisco.reyes@estudiantes.cicy.mx (F.A.R.-S.); ubt_fguillen@cicy.mx (F.G.-C.); mianheal@cicy.mx (M.A.H.-A.); 2 Integrative Biology Department, Yucatán Scientific Research Center A.C., Mérida CP 97205, Yucatán, Mexico; enriquec@cicy.mx (E.C.d.l.S.); cicy.santey@gmail.com (L.F.S.-T.); 3 Secretaria de Ciencia, Humanidades, Tecnología e Innovación (SECIHTI)-Research and Assistance Center in Technology and Design of the State of Jalisco, Parque Científico Tecnológico de Yucatán, Mérida CP 97302, Yucatán, Mexico

**Keywords:** tomato, disordered regions, proteomics, bioinformatics

## Abstract

Protein identity and functional roles within the cell provide the landscape of proteomics and other high-throughput technologies. However, not all protein sequences are cataloged with an identity or a functional protein family. The lack of identity and functional role of a set of proteins are collectively named as the dark proteome. Key structural features are, for example, ordered sequences (with a defined structural arrangement) and disordered sequences (presenting one or more intrinsically disordered stretches). Here, we reanalyzed eight proteomic datasets and the subset of the “unknown” proteome of *S. lycopersicum* to describe if there is a relationship between disorder, length, and tissue-specific abundance of proteins with key structural features in the relation of ordered/disordered abundance in the protein sequences. Intriguingly, we unveil that from the *S. lycopersicum* proteome, the “unknown” subset represents around 10% only. We further cataloged dark proteome in terms of ordered and disordered sequences and found that proteins with disorder represent around 23% of the total “unknown” proteins. Also, we describe an amino acid composition and sequence length enrichment both, in the ordered and disordered fraction of the dark proteome. Finally, we describe that proteins within the dark proteome can be related to a specific location and abundance in an organ or tissue. An unknown protein sequence presenting a combination of specific length and degree of disorder can be explored with other biotechnological alternatives to improve responses or tolerate abiotic stress, also serving as sensors during development or ripening stages. These findings suggest an opportunity to study “protein darkness” in terms of disorder and functional associations.

## 1. Introduction

Crop production is constantly challenged by environmental stresses, impacting yield and quality. Understanding the molecular mechanisms underlying stress responses is crucial for developing resilient crops. The so-called “omics” technologies can help us to uncover key regulatory mechanisms and responses to these stresses in plant science. Among them, proteomics plays a key role in deciphering these mechanisms. However, the presence of unassigned protein sequences, i.e., “dark proteome”, hinders comprehensive analysis, especially in non-model crops. *Solanum lycopersicum* L. serves as a crucial model for studying fruit development, particularly in climacteric fruits [1,2,3,4,5,6]. Its sequenced genome (950 Mb, 12 chromosomes) and well-established transformation tools make it an ideal system to evaluate the physiological and molecular relationship between plant responses and stress [7,8]. Current data from emerging research in tomato have been deposited in several databases, for example, the International Tomato Annotation Group (iTAG) project with 34,727 coding genes annotated (iTAG v2.3), and intriguingly ~7640 sequences remained as “unknown proteins” [7], representing the dark proteome in tomato. Updated iTAG versions (v4.0 and 4.1) continue to contain dark sequences, highlighting the need for their characterization [9,10]. For example, in the iTAG (4.1) database around 3801 sequences are annotated as “Unknown proteins”.

Proteomics, also known as a large-scale study of the entire protein complement (proteome) of a biological sample under specific conditions, provides a dynamic view of protein representation [11,12]. The concept of “dark proteome” in proteomics comprises protein sequences with limited functional and/or structural information. Protein domains are defined both by their capacity for independent folding (structural definition) and by evolutionary conserved amino acid sequences (sequence-based definition) [13,14]. Despite the existence of domain databases, sequence annotation remains incomplete, particularly for non-model organisms and orphan sequences [15,16,17,18]. Similarity criteria are employed for annotation, but sequences that do not meet these criteria constitute the dark proteome. This is further categorized into dark proteins (completely unannotated), dark regions (unannotated segments within a protein), PDB or white protein/regions (with structural information), and gray regions (annotated by inference). Several intrinsically disordered proteins (IDPs) and transmembrane proteins are prominent examples of complex dark proteins, but do not represent the full dataset of a dark proteome [13,19,20,21]. Key protein databases include UniProt (sequences) and the Protein Data Bank (PDB; 3D structures). However, with over 65 million sequences in UniProt and approximately 125,000 structures in the PDB, less than 0.1% of UniProt sequences have experimentally determined 3D structures [22].

Homology modeling allows for structure inference for approximately half of the remaining sequences [23]. Previous studies have quantified the dark proteome across various taxa, revealing a high proportion of gray regions and variable proportions of dark proteins [19]. Hydrophobic topology analysis, such as hydrophobic cluster analysis (HCA), enables the identification of foldable regions even without homologous sequence information [13]. Studies in model organisms, including *Arabidopsis thaliana*, *Caenorhabditis elegans*, *Escherichia coli*, *Saccharomyces cerevisiae*, and *Mus musculus*, reveal a significant proportion of the proteome residing in the dark, often with specific subcellular localizations [21].

While the terms dark proteome and IDPs are sometimes used interchangeably, they are not synonymous. In fact, IDPs are a subset of the dark proteome, which also includes proteins with HCA domains and orphan sequences [20,24]. IDPs, however, have garnered significant attention due to their involvement in plant stress responses. Their structural disorder confers a crucial role in transcriptional regulation, particularly in transcription factors (TFs), where intrinsically disordered regions (IDRs) are abundant in activation domains [25,26]. Examples of plant protein families with relevant IDRs include GRAS [27,28], cryptochromes (CRYs) [29,30], and late embryogenesis abundant (LEA) proteins, including dehydrins [31,32,33].

Here, with this work, we focused on the quantification and characterization of the subset of IDP abundance in the dark proteome of *S. lycopersicum* iTAG 4.1, aiming to assign functional annotations and contribute to a better understanding of biological processes in this important crop, serving as a starting line for the development of novel strategies to mitigate or tolerate stress and understand the roles of proteins without an assigned functionality. To date, no previous works have described the abundance of disorder and dark proteomein in a tissue-specific manner and in the vascular system of non-model plants, like tomato. Although seminal work from Perdigão has increased our understanding about dark proteome, more information is need in order to understand their role and possible application in crop management [19,20,21]. Our results describe the relationship between length, abundance, and IDR prediction based on reanalyzed proteomic data from public databases, unveiling how and when uncharacterized proteins (UPs) can be modulated to cover functions and molecular processes in the plant.

## 2. Materials and Methods

### 2.1. Data Acquisition of S. lycopersicum Protein Sequences

Tomato protein sequences were downloaded in FASTA format from the Sol Genomics Network FTP site, version iTAG4.1 (https://solgenomics.net/ftp//tomato_genome/annotation/ITAG4.1_release/, data retrieved on 10 November 2024) [7]. Sequences described as “unknown protein” (UP) were selected to form the iTAG4.1 dark protein database. We highlighted the convenience of using this term, but we stated that UP corresponds to a part of all the dark proteome. Supplementary Table S1 condenses the data ID repository in the ProteomeXchange database (https://proteomecentral.proteomexchange.org/, accessed on 10 November 2024) [34]; the column titled “Proteomic data acquisition” depicts the proteomic approach used for each study. The datasets were selected based primarily on the following criteria: (a) shotgun and label-free LC-MS/MS quantitative proteomics, (b) tissue-specific and related to xylem, phloem, or pollen grains, (c) subjected to abiotic stress and healthy conditions.

### 2.2. Further Classification and Curation of up Dark Protein Database from S. lycopersicum

Retrieved protein sequences of *S. lycopersicum* from the SolGenomics Network were subjected to further processing, trying to assign identity to all classified as UP. This was achieved by a two-approach analysis. First, a local BLASTP [35] was performed with the UP dataset against the Plant Protein Sequences (PPS) database from the Uniprot Database (https://www.uniprot.org/, released version 2024-01, accessed on 3 April 2025). The parameters for this BLASTP were (1) one hit per sequence, (2) an e-value threshold of 1 × 10^−10^, and (3) sequence coverage > 50%. All sequences with at least ~50% of identity and a *p*-value of 1 × 10^−10^ were removed from the UP dataset. Second, a Pfam analysis [36] was carried out in order to find family domains, following the command line: /interproscan.sh -appl Pfam -i/path/to/sequences_dark_proteome.fasta, with an e-value of 0.001 and a domain E value (-domE) threshold of 0.001.

### 2.3. Intrinsically Disordered Prediction of up Dataset

In order to predict a functional subset fraction of UP dataset, intrinsically disordered prediction was carried out with flDPnn V2.0 [37] and MobiDB-lite 3.0 [38]. Briefly, flDPnn is a neural deep network developed to provide accurate, fast, and comprehensive disordered regions and proteins based on amino acid sequences. FlDPnn provides predictions in accordance with the Critical Assessment of Intrinsic Disorder Prediction (CIAD) [39]. Moreover, the prediction includes a propensity of functions frequently related to IDRs; DNA and RNA binding, linker sequences, and protein binding. On the other hand, MobiDB-lite provides a set of complementary ID predictors and an optimized consensus of PDB X-ray data to ensure a correct prediction of different classes of disorder types. Curated *S. lycopersicum* dataset from previous step was used with both predictors. For both algorithms the limit threshold was ≥0.5 to consider as sequences with propensity to disorder.

### 2.4. Reanalysis of S. lycopersicum Mass Spectra

Mass spectra corresponding to eight experiments (sets) of *S. lycopersicum* under different conditions were downloaded from the public and open access ProteomeXchange repository (http://www.proteomexchange.org, accessed on 15 April 2025), as described in Supplementary Table S1 (ST1). Each of the eight datasets was reanalyzed using the MaxQuant software [40] v2.0 in label-free quantification (LFQ) mode [41] with preset parameters and the FragPipe v23.1 software with the MSFragger v4.1 search engine [42] in preset label-free quantification mode.

### 2.5. Proteomic Data Processing and Visualization

The number of proteins identified by each tool was visualized using a bar chart. A Venn diagram was used to illustrate the overlap in protein identifications between the tools. The distribution of proteins across different experimental sets (*n* = 8) for each tool was visualized using a stacked bar chart. The number of proteins within each category was displayed within each segment of the bars. To explore the intersections and unique elements within the different experimental sets, an UpSet plot was generated using the UpSetR package (v1.4.0). This visualization displayed the size of intersections between the sets, allowing for the identification of shared and unique proteins across the different experimental conditions. For bar charts and Venn diagrams, the ggplot2 package (version 3.5.2) was used in the R Studio with R statistical language (version 4.4.1). For clustering analysis, the data were scaled using the scale function in R. The optimal number of clusters for partitioning around medoids (PAM) was determined using the fviz_nbclust function (factoextra package, v1.0.7) with the silhouette method. This function evaluates the clustering performance across a range of cluster numbers (*k* = 1 to 10) using the CLARA algorithm for efficient handling of potentially large datasets. The average silhouette width, measuring cluster cohesion and separation, was calculated for each *k* across 100 bootstrap samples. The *k* maximizing the average silhouette width, indicating the most stable cluster configuration, was selected. Based on this analysis, PAM clustering was performed using the pam function (cluster package) with the identified optimal *k* value (7 in this case). The resulting clusters were visualized using fviz_cluster (factoextra package, v1.0.7), displaying the clustering in the first two principal components. A network representation of the data were generated using Cytoscape v3.10.3.

## 3. Results

### 3.1. Identification, Processing, and Curation of Dark Proteins in S. lycopersicum and Their Functional Assesment into Ordered and Disordered Regions

The tomato proteome comprises 34,689 sequences, of which 3797 were classified as unknown proteins (UPs). These proteins formed the UPDB (Uncharacterized Protein Data Bank), a local dedicated database for the collection and storage of information on uncharacterized proteins. From BLASTP results (Supplementary File S3) and PFAM results (Supplementary File S4), further annotation of protein sequences was carried out from our UP dataset. A total of 841 protein sequences were assigned with identity in both tools used. These sequences were removed from starting dataset and the final curated UP dataset with 3377 protein sequences was used to all the following analysis. This step has been crucial for expanding and enriching the knowledge of dark proteins in the specific context of tomato, thus providing a more complete and detailed view of its protein composition.

**Figure 1:**
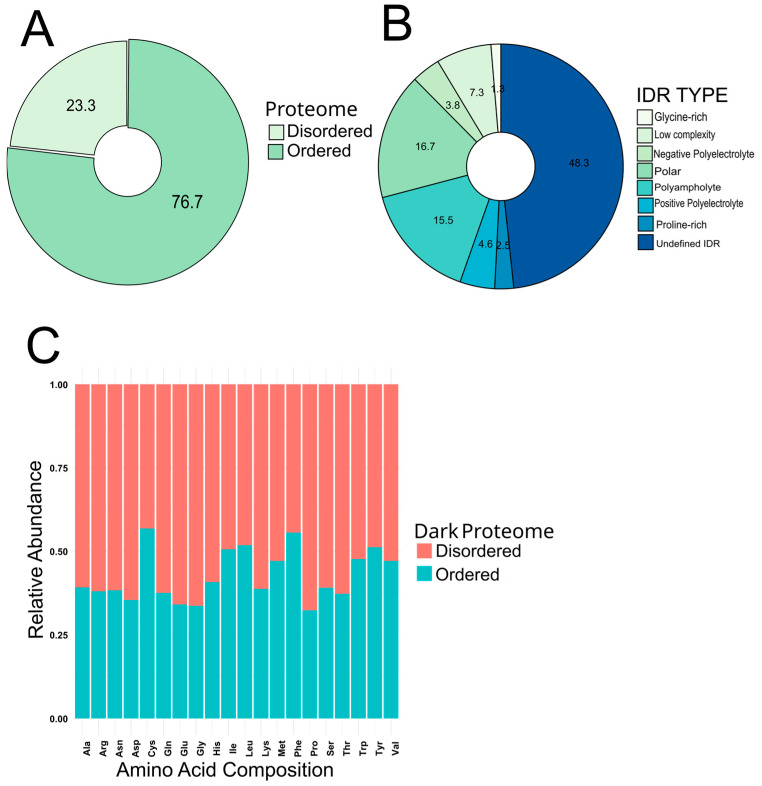
Distribution of ordered and disordered protein sequences into the dark proteome of *S. lycopersicum*. (**A**) Proportional comparison of the abundance of ordered and disordered proteins. (**B**) Distribution of different IDP identified in the disordered dark proteome. (**C**) Amino acid composition comparison between ordered and disordered dark proteome.

**Figure 2:**
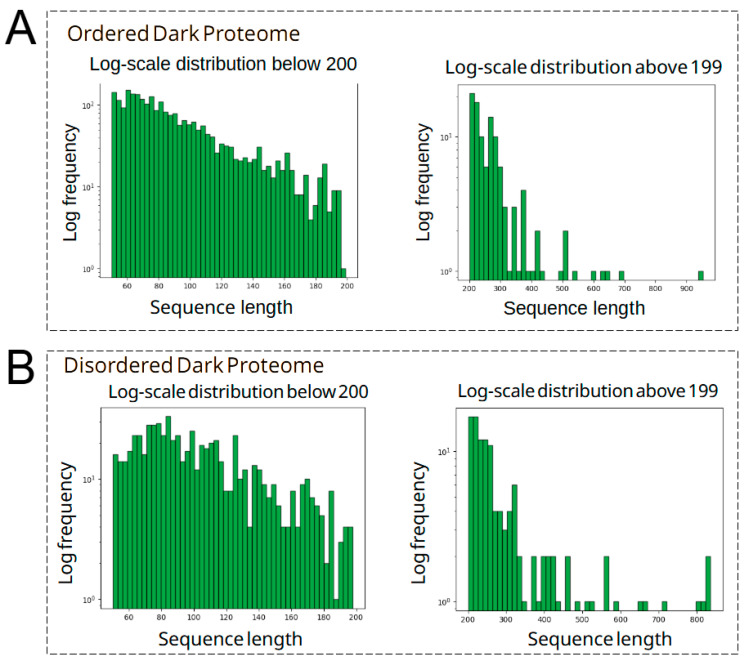
Distribution of the ordered and disordered dark proteome based on length. (**A**) Amino acid length distribution of the ordered proteins presented in a log10 value. (**B**) Amino acid length distribution of the disordered proteins presented in a log value. The amino acid sequences were set up with 200 aa in length as an arbitrary threshold to compare their abundances.

From our analysis with flDPnn and MobiDB-lite software we were able to predict, identify, and quantify amino acid composition of the dark proteome of *S. lycopersicum*. A total of 786 sequences at least 50 aa in length were predicted to possess IDR or to be IDP. This represents about ~23% of the total of protein sequences with an unknown function and identity described here (Figure 1A). Proteins with no intrinsically disordered (ID) prediction were cataloged as ordered proteins. Moreover, our analysis can also classify the intrinsically disordered regions into several types. Undefined ID regions were the more abundant with 48.3% of the total of IDR predicted, with polar (16.7%), polyampholyte (15.5%), the low complexity group with 7.3%, and positive polyelectrolyte with 4.6% noted as the top five identified ID regions composing the dark proteome analyzed (Figure 1B). Also, the amino acid composition was evaluated in the ordered and the disordered dark proteome, with an enrichment of Ala, Arg, Asn, Asp, Gln, Gly, Glu, His, Lys, Pro, Ser, and Thr aa with more than 50% of their abundance within the disordered proteome (Figure 1C). Moreover, we investigated the proportional abundance of proteins based on aa length constituting the ordered and disordered dark proteome. Both ordered and disordered dark proteome possess most of its sequences in the order of 200 aa, with 95% (2483 sequences) and 85% (671 sequences), respectively. These show a similar distribution of both structural configurations. A small subset of proteins in both dark proteomes with an interval length within 200–300 aa were identified with 85 sequences and 80 sequences in the ordered and disordered datasets, respectively (Figure 2).

### 3.2. Experimental Identification of Dark Proteins and Network Analysis of Dark Proteins

**Figure 3:**
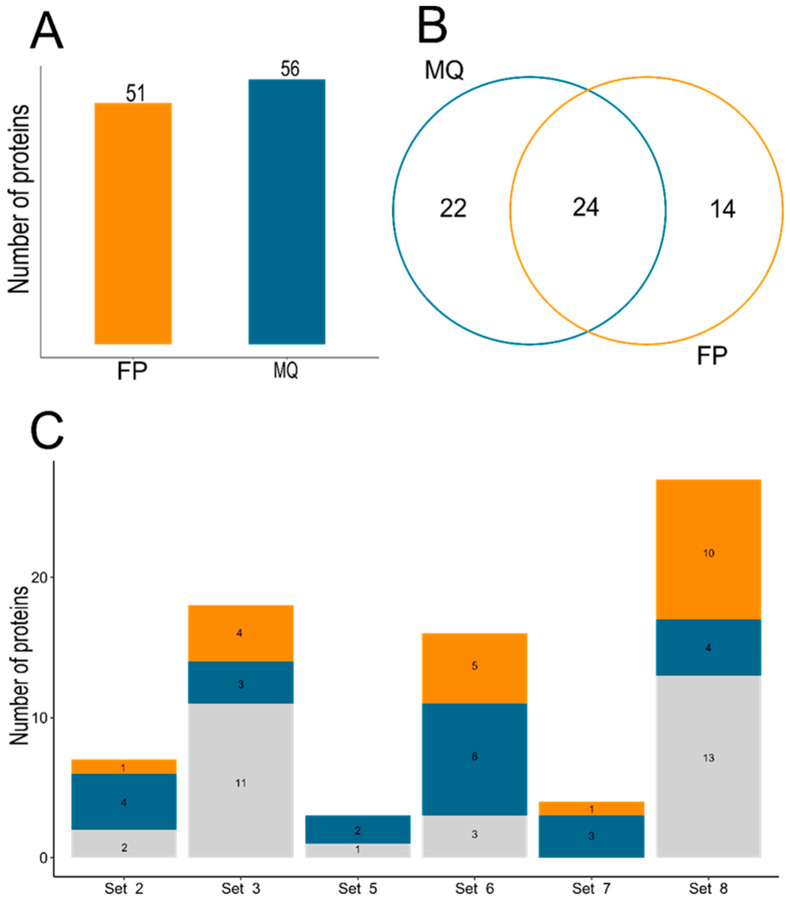
Identification of the dark proteome through a reanalyzed data. (**A**) Bar plot. Number of UPs detected by MaxQuant (MQ) (blue) and FragPipe (FP) (orange). The highest number of UPs detected was 56, by MaxQuant. (**B**) Venn diagram. Shared and unique UPs between bioinformatics tools. Both tools identified 24 UPs in common, while 22 and 14 unique UPs were identified for each bioinformatics tool, respectively. (**C**) Stacked bar plot. UPs identified by MQ, FP, and both in each dataset. Datasets 8 and 3 achieved the highest number of UPs identified, respectively. In both, the shared UPs, i.e., those identified by both tools, are greater than the number of UPs uniquely identified by each tool. Color code was used as follows: FragPipe in orange and MaxQuant in blue.

**Figure 4:**
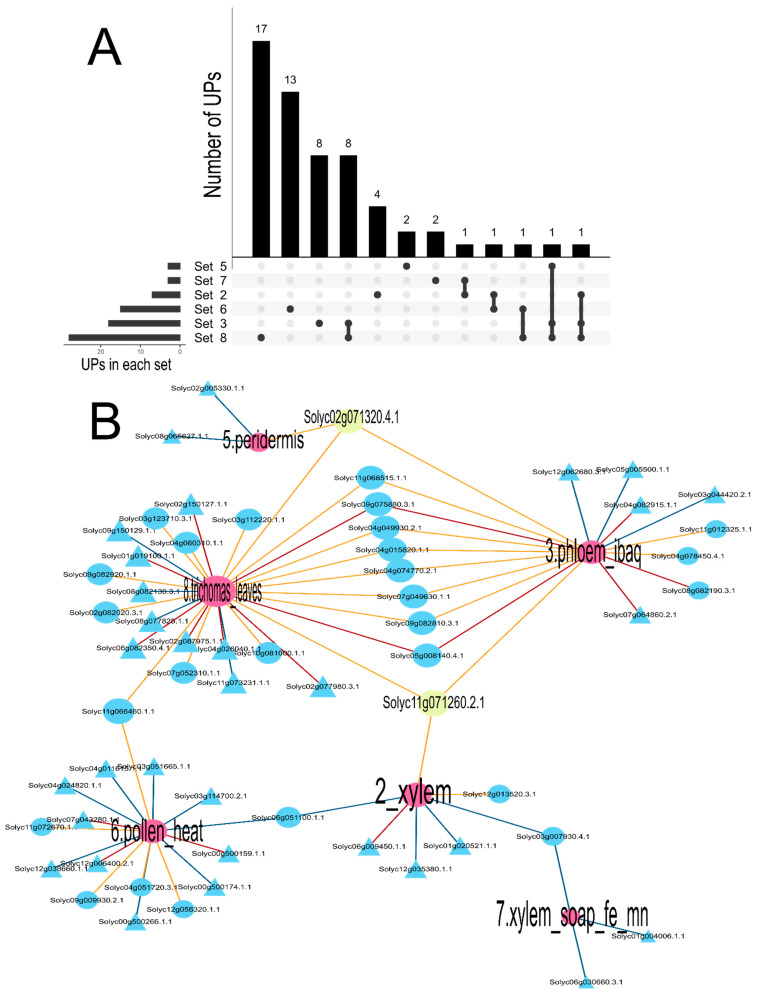
Abundance of UPs was identified based on the dataset used in this study. (**A**) Upset plot with shared and unique UPs between datasets are represented in the plot. Dataset 6 recorded more unique UPs with MaxQuant (7 UPs), while for dataset 8 unique sequences obtained with Fragpipe was 6 Ups. In the lower panel the black dots represent unique protein sequences identified by MaxQuant or FragPipe, and black lines represent the intersection of protein sequences identified by both methods used, between datasets. (**B**) Network visualization of proteins in tomato proteomic datasets. Blue nodes represent proteins, yellow nodes highlight the most shared proteins between datasets (Solyc11g071260.2.1, Solyc02g071320.4.1). Red nodes connecting proteins to their corresponding datasets are depicted.

To experimentally identify dark proteins, the FragPipe and MaxQuant software were used to reanalyze eight datasets obtained from the ProteomeXchange repository. MaxQuant detected 56 UPs, while FragPipe identified 51 (Figure 3A). Of these proteins, both tools coincided in the identification of 24 UPs, but MaxQuant identified 22 unique UPs and FragPipe 14 (Figure 3B). MaxQuant identified UPs in six of the eight datasets; sets 1 and 4 showed no Ups. On the other hand, FragPipe detected UPs in five sets and three sets showed no Ups; sets 1, 4, and 7 (Figure 3C). Details of the UPs per set and tool can be found in Supplementary Table S2 (ST2). An UpSet plot was used to visualize the UPs detected by MaxQuant and FragPipe, including unique UPs (Figure 4A). Set 6 recorded the highest number of unique UPs with MaxQuant (7 UPs), while set 8 did so with FragPipe (6 UPs). Regarding UPs detected in multiple sets, only one was present in all, except for set 8 in FragPipe. The visualization of shared and unique UPs per set was performed using Cytoscape (Figure 4B).

Figure 4B presents a network visualization exploring the presence of proteins in tomato proteomic datasets. Blue nodes represent individual proteins, while edges connect these proteins to tomato datasets (red nodes), indicating that the protein was identified in that specific proteomic dataset. In particular, yellow nodes highlight the most shared tomato UPs between sets: Solyc11g071260.2.1 and Solyc02g071320.4.1. Solyc11g071260.2.1 was identified in datasets related to xylem, phloem, and leaf trichomes, while Solyc02g071320.4.1 was found in datasets related to phloem, periderm, and leaf trichomes. The presence of these UPs in multiple datasets suggests that they may be involved in diverse biological processes or expressed in multiple tissues within the plant.

### 3.3. Identification of Intrinsically Disordered Proteins in the Vascular Transport System of S. lycopersicum

**Figure 5:**
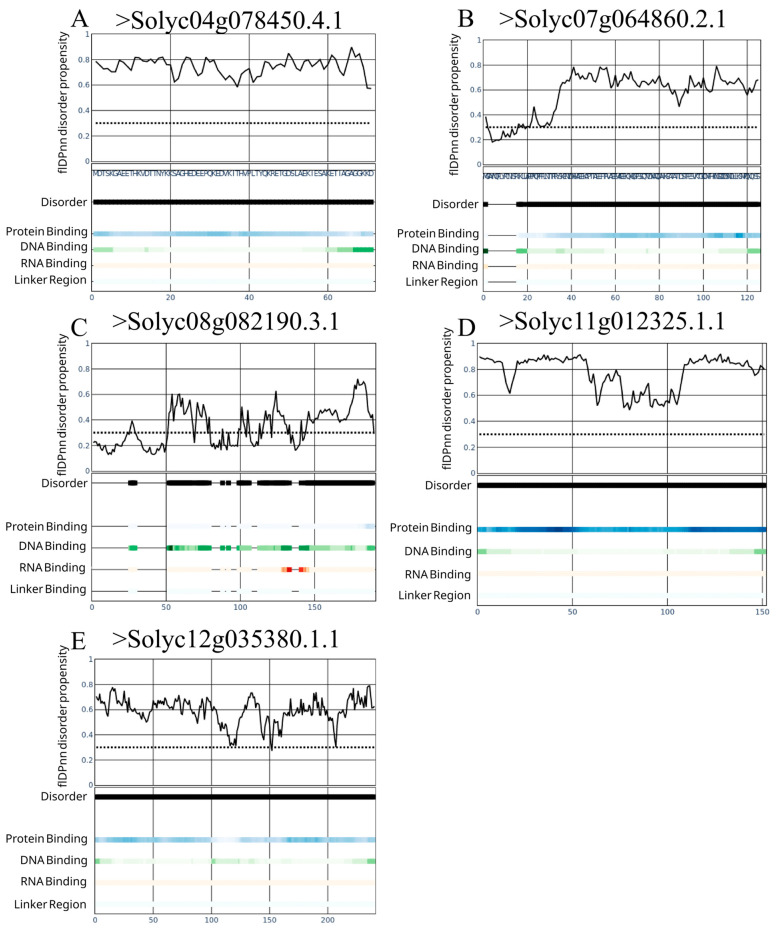
Intrinsically disordered proteins identified in phloem and xylem. (**A**–**E**) UP’s identifiers and their tendency to disorder, DNA/RNA binding, and protein–protein interaction. Color code was used to describe protein binding in blue, DNA binding in green, RNA binding with red and liker regions with gray scale. Dotted line represents the 0.3 threshold above which proteins exhibit propensity to disorder.

We wondered if there is an enrichment of specialized protein structure, with an ordered or disordered propensity present in the local and vascular transport system. Datasets 2, 3, and 7, covering xylem and phloem sap, were used after MaxQuant and FragPipe identification. A total of 14 protein sequences were identified to be present both in the xylem and phloem. Nine proteins with ordered sequences and five with disorder prediction were identified (Figure 5). From the flDPnn potential functional categorization we uncovered that none of the proteins identified at the xylem and phloem possess RNA binding regions and only >Solyc08g082190.3.1 possessed a high tendency to interact with DNA. Protein–protein interactions were identified in the >Solyc11g012325.1.1 sequence. Four of the five proteins were predicted to have high tendency to disorder at full length level (Figure 5A,B,D,E). Intriguingly, only >Solyc08g082190.3.1 sequences possessed at least four regions with a high tendency to disorder, but no clear potential functional behavior was predicted (Figure 5C).

### 3.4. Clustering Analysis of Dark Proteins

**Figure 6:**
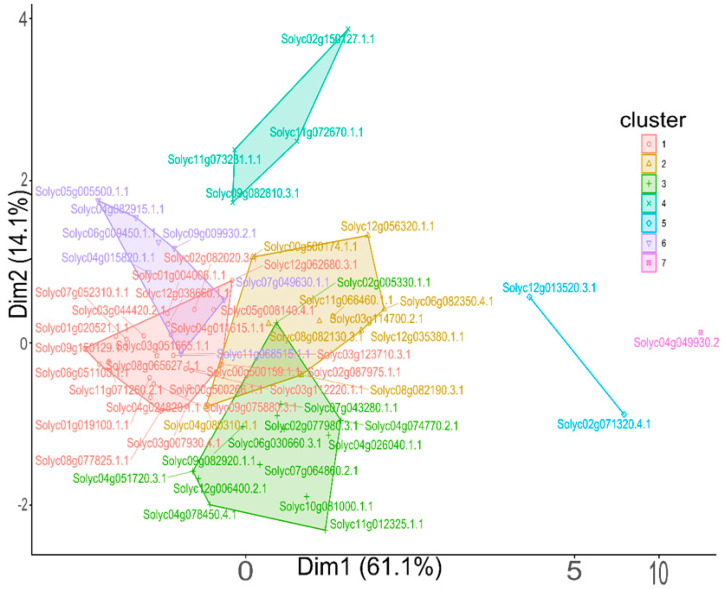
A multiple-component analysis of UP. Proteins (*n* = 60) are colored by cluster (*n* = 7) according to features including molecular weight, pI, GRAVY score, net charge, amino acid counts, and predicted TM segments. Code color depicts the number of clusters.

To explore potential relationships among the identified UPs, we performed principal component analysis (PCA) based on their molecular and biochemical properties, including molecular weight, isoelectric point (pI), hydrophobicity (GRAVY score), net charge, amino acid composition, and predicted transmembrane helices. PCA reduced the data dimensionality, revealing five distinct clusters of UPs (Figure 6). Notably, Solyc04g049930.2.1 formed a unique cluster, suggesting it possesses distinct characteristics compared to the other UPs. The clustering suggests potential functional or structural similarities among UPs within each group.

## 4. Discussion

This study aimed to shed light on the “dark proteome” of tomato by identifying and characterizing uncharacterized proteins (UPs) in several tissues and vascular system of tomato. Our analysis revealed a significant number of UPs within the tomato proteome, highlighting the substantial gap in our current understanding of protein function in this important crop. The percentage of uncharacterized proteins (UPs) in the tomato proteome (10.95%) is notably lower than that reported for *Arabidopsis* (41.2%) by Perdigão and Rosa (2019) [21]. This suggests that a smaller proportion of the tomato proteome remains functionally uncharacterized. This discrepancy may be attributed to several factors, including inherent species-specific differences in genome size, gene duplication events, and evolutionary history. Additionally, differences in the completeness and accuracy of genome and transcriptome sequencing and annotation efforts between the two species could significantly influence the predicted proteome size and the proportion of uncharacterized proteins. The initial step of creating the UPDB (Uncharacterized Protein Data Bank) was crucial. This repository serves as a valuable resource for the tomato research community, facilitating further investigation and characterization of these enigmatic proteins. By systematically collecting and storing information on UPs, we have expanded the knowledge base of the tomato proteome, paving the way for a more comprehensive understanding of its biological complexity. The reanalysis of publicly available proteomic datasets using two independent software tools (FragPipe v23.1 and MaxQuant v4.1) provided valuable insights into the experimental identification of UPs. While both tools identified a significant number of UPs, the observed discrepancies in their results underscore the challenges associated with confidently identifying and quantifying low-abundance or novel proteins. The identification of unique UPs by each tool highlights the importance of employing multiple analytical approaches to maximize the coverage of the proteome. The network analysis revealed interesting patterns in the distribution of UPs across different tomato tissues and developmental stages. The observation that Solyc11g071260.2.1 is found in xylem, phloem, and leaf trichomes, and Solyc02g071320.4.1 is present in phloem, periderm, and leaf trichomes, strongly suggests these UPs play roles in fundamental cellular processes common to these diverse tissues. Despite their distinct functions—water and mineral transport in xylem [43], sugar transport in phloem [44], and specialized roles like defense and water regulation in trichomes [45]—these tissues share essential cellular needs. These include energy production for cellular activities, responses to various environmental stresses, and maintaining structural integrity [46,47]. Furthermore, periderm, as the outer protective layer, shares protective functions with leaf trichomes [48]. This broad distribution of these UPs across these diverse tissues suggests their involvement in essential housekeeping functions, such as cellular metabolism, signal transduction, and maintaining cellular homeostasis, as well as in general plant stress responses. Here also, we highlight the limitations of this study, regarding the distribution and abundance of UP, which represents a subset of the current knowledge of dark proteomes. For example, other omics technologies coupled with proteomics, as transcriptomics, can unveil transcript abundance of specific UPs across tissue types and stresses. Moreover, other authors have described more details about the concept and classification of dark proteome, depending on its origin as (a) short ORFs, or (b) upstream ORFs, non-canonical ORFs, dark regions, and IDRs [13,19,49]. Due to the lack of a unified classification of proteins without a functionality assignment, the study of dark proteome in terms of disorder represents an initial step for further analysis, description, and abundance of this subset of proteins present in several organisms.

Uncovering the distribution of the dark proteome of *S. lycopersicum* in terms of disorder and order reveals that the order/disorder fraction are substantial for IDPs, at around 23%, suggesting that IDPs represents an important fraction of the dark proteome, as previously suggested by other authors [50,51,52,53]. ID regions and proteins are frequently related to response to abiotic stressors like desiccation, flooding, heat, cold, and salinity [54,55,56]. Probably, its rapid conformational change and molecular propensity to bind to other proteins and DNA/RNA can serve as signaling molecules [57,58]. This is accomplished by an enrichment of amino acids that favors the promiscuity of IDP, such Gly, Gln, Ar, Lys, and Pro amino acids [59], which have the potential to drive multiple transitory interactions. Also, length is a key factor associated with IDP, as observed in our results; most of the disordered dark proteome consists of sequences under 200 aa in length, suggesting that length could confer plasticity and a rapid response to other molecules and clues from abiotic stresses [26,60]. The observation about the presence of intrinsically disordered UP in xylem and phloem could shed some light about the functional role of this type of protein. For example, phloem transport and long-distance movement of macro-molecules have been discussed to be energetically demanding processes or passive non-demanding energy [61,62]. Phloem also transports other molecules different to proteins, particularly RNA. This suggested that efficient transport and preventing RNA degradation should be due to the formation of ribonucleo particles consisting of RNA-Protein complexes. However, our identified sequences from phloem datasets identifies IDP without a high tendency to interact with DNA or RNA. On the other hand, IDP has been suggested to bind RNA cooperatively. This intriguing result could indicate other functions conducted by IDP present in the phloem.

Finally, the PCA based on various physicochemical properties grouped UPs into distinct clusters. The formation of unique clusters, such as that observed for Solyc04g049930.2.1, suggests that these proteins possess distinct characteristics, potentially reflecting unique functional roles or structural features. The clustering analysis provides valuable insights into potential functional or structural relationships among UPs, which can guide future experimental investigations.

## 5. Conclusions

This study significantly advances our understanding of the tomato proteome by identifying and characterizing a substantial number of previously uncharacterized proteins (UPs). While relying on existing proteomic datasets presents inherent limitations due to variations in experimental conditions and data processing, this study provides a crucial foundation for future investigations. Future research should prioritize the functional characterization of these identified UPs. This includes employing techniques such as gene expression analysis, protein–protein interaction studies, and mutant analysis to elucidate their biological functions. Additionally, determining the subcellular localization and three-dimensional structures of these proteins will provide valuable insights into their molecular mechanisms.

In conclusion, this study represents a significant step towards a more complete and accurate representation of the tomato proteome. The findings have broad implications for understanding plant growth, development, and stress responses, paving the way for future research aimed at improving crop productivity and resilience.

## Data Availability

All data generated or analysed during this study are included in the published article.

## Supplementary Materials

The following supporting information can be downloaded at: https://www.mdpi.com/article/10.3390/cimb47090769/s1.

## Abbreviations

The following abbreviations are used in this manuscript:

UP	Unknown Protein
IDP	Intrinsically Disordered Proteins
IDR	Intrinsically Disordered regions
TF	Transcription Factors
CRY	Criptochormes
LEA	Late Embryogenesis Abundant
LFQ	Label-Free Quantification
UPDB	Uncharacterized Protein Data Bank

## References

[1] Sant’Ana D.V.P., Lefsrud M. (2018). Tomato proteomics: Tomato as a model for crop proteomics. Sci. Hortic..

[2] Gerszberg A., Hnatuszko-Konka K., Kowalczyk T., Kononowicz A.K. (2015). Tomato (*Solanum lycopersicum* L.) in the service of biotechnology. Plant Cell Tissue Organ Cult..

[3] Karlova R., Chapman N., David K., Angenent G.C., Seymour G.B., de Maagd R.A. (2014). Transcriptional control of fleshy fruit development and ripening. J. Exp. Bot..

[4] Kimura S., Sinha N. (2008). Tomato (*Solanum lycopersicum*): A Model Fruit-Bearing Crop. Cold Spring Harb. Protoc..

[5] Doganlar S., Frary A., Tanksley S.D. (2000). The genetic basis of seed-weight variation: Tomato as a model system. Theor. Appl. Genet..

[6] Alexander L., Grierson D. (2002). Ethylene biosynthesis and action in tomato: A model for climacteric fruit ripening. J. Exp. Bot..

[7] Rombauts S., Causse M., Giovannoni J., Bouzayen M., Zouine M. (2016). Annotation of the Tomato Genome. The Tomato Genome.

[8] The Tomato Genome Consortium (TGC) (2012). The tomato genome sequence provides insights into fleshy fruit evolution. Nature.

[9] Hosmani P.S., Flores-Gonzalez M., van de Geest H., Maumus F., Bakker L.V., Schijlen E., van Haarst J., Cordewener J., Sanchez-Perez G., Peters S. (2019). An improved de novo assembly and annotation of the tomato reference genome using single-molecule sequencing, Hi-C proximity ligation and optical maps. bioRxiv.

[10] Fernandez-Pozo N., Menda N., Edwards J.D., Saha S., Tecle I.Y., Strickler S.R., Bombarely A., Fisher-York T., Pujar A., Foerster H. (2015). The Sol Genomics Network (SGN)—From genotype to phenotype to breeding. Nucleic Acids Res..

[11] Gautam A., Pandey P., Pandey A.K., Tripathi D.K., Pratap Singh V., Chauhan D.K., Sharma S., Prasad S.M., Dubey N.K. (2020). Chapter 20—Proteomics in relation to abiotic stress tolerance in plants. Plant Life Under Changing Environment.

[12] Griffiths W.J., Wang Y. (2009). Mass spectrometry: From proteomics to metabolomics and lipidomics. Chem. Soc. Rev..

[13] Bitard-Feildel T., Callebaut I. (2017). Exploring the dark foldable proteome by considering hydrophobic amino acids topology. Sci. Rep..

[14] Finn R.D., Coggill P., Eberhardt R.Y., Eddy S.R., Mistry J., Mitchell A.L., Potter S.C., Punta M., Qureshi M., Sangrador-Vegas A. (2016). The Pfam protein families database: Towards a more sustainable future. Nucleic Acids Res..

[15] Nepomnyachiy S., Ben-Tal N., Kolodny R. (2014). Global view of the protein universe. Proc. Natl. Acad. Sci. USA.

[16] Faure G., Callebaut I. (2013). Comprehensive Repertoire of Foldable Regions within Whole Genomes. PLoS Comput. Biol..

[17] Tautz D., Domazet-Lošo T. (2011). The evolutionary origin of orphan genes. Nat. Rev. Genet..

[18] Caetano-Anollés G., Caetano-Anollés D. (2003). An Evolutionarily Structured Universe of Protein Architecture. Genome Res..

[19] Perdigão N., Heinrich J., Stolte C., Sabir K.S., Buckley M.J., Tabor B., Signal B., Gloss B.S., Hammang C.J., Rost B. (2015). Unexpected features of the dark proteome. Proc. Natl. Acad. Sci. USA.

[20] Perdigão N., Rosa A.C., O’Donoghue S.I. (2017). The Dark Proteome Database. BioData Min..

[21] Perdigão N., Rosa A. (2019). Dark Proteome Database: Studies on Dark Proteins. High-Throughput.

[22] Berman H.M., Westbrook J., Feng Z., Gilliland G., Bhat T.N., Weissig H., Shindyalov I.N., Bourne P.E. (2000). The Protein Data Bank. Nucleic Acids Res..

[23] Scaiewicz A., Levitt M. (2018). Unique function words characterize genomic proteins. Proc. Natl. Acad. Sci. USA.

[24] Woodcock S., Mornon J.P., Henrissat B. (1992). Detection of secondary structure elements in proteins by hydrophobic cluster analysis. Protein Eng..

[25] Zamora-Briseño J.A., Pereira-Santana A., Reyes-Hernández S.J., Cerqueda-García D., Castaño E., Rodríguez-Zapata L.C. (2021). Towards an understanding of the role of intrinsic protein disorder on plant adaptation to environmental challenges. Cell Stress Chaperones.

[26] Covarrubias A.A., Romero-Pérez P.S., Cuevas-Velazquez C.L., Rendón-Luna D.F. (2020). The functional diversity of structural disorder in plant proteins. Arch. Biochem. Biophys..

[27] Choura M., Ebel C., Hanin M. (2019). Genomic analysis of intrinsically disordered proteins in cereals: From mining to meaning. Gene.

[28] Sun X., Jones W.T., Rikkerink E.H.A. (2012). GRAS proteins: The versatile roles of intrinsically disordered proteins in plant signalling. Biochem. J..

[29] Kang C.Y., Lian H.L., Wang F.F., Huang J.R., Yang H.Q. (2009). Cryptochromes, Phytochromes, and COP1 Regulate Light-Controlled Stomatal Development in Arabidopsis. Plant Cell.

[30] Li Q.H., Yang H.Q. (2007). Cryptochrome Signaling in Plants. Photochem. Photobiol..

[31] Yu Z., Wang X., Zhang L. (2018). Structural and Functional Dynamics of Dehydrins: A Plant Protector Protein under Abiotic Stress. Int. J. Mol. Sci..

[32] Banerjee A., Roychoudhury A. (2016). Group II late embryogenesis abundant (LEA) proteins: Structural and functional aspects in plant abiotic stress. Plant Growth Regul..

[33] Kovacs D., Agoston B., Tompa P. (2008). Disordered plant LEA proteins as molecular chaperones. Plant Signal. Behav..

[34] Deutsch E.W., Bandeira N., Perez-Riverol Y., Sharma V., Carver J.J., Mendoza L., Kundu D.J., Wang S., Bandla C., Kamatchinathan S. (2023). The ProteomeXchange consortium at 10 years: 2023 update. Nucleic Acids Res..

[35] Altschul S.F., Gish W., Miller W., Myers E.W., Lipman D.J. (1990). Basic local alignment search tool. J. Mol. Biol..

[36] Paysan-Lafosse T., Andreeva A., Blum M., Chuguransky S.R., Grego T., Pinto B.L., Salazar G.A., Bileschi M.L., Llinares-López F., Meng-Papaxanthos L. (2025). The Pfam protein families database: Embracing AI/ML. Nucleic Acids Res..

[37] Hu G., Katuwawala A., Wang K., Wu Z., Ghadermarzi S., Gao J., Kurgan L. (2021). flDPnn: Accurate intrinsic disorder prediction with putative propensities of disorder functions. Nat. Commun..

[38] Necci M., Piovesan D., Clementel D., Dosztányi Z., Tosatto S.C.E. (2021). MobiDB-lite 3.0: Fast consensus annotation of intrinsic disorder flavors in proteins. Bioinformatics.

[39] Necci M., Piovesan D., Tosatto S.C.E., CAID Predictors, DisProt Curators (2021). Critical assessment of protein intrinsic disorder prediction. Nat. Methods.

[40] Cox J., Mann M. (2008). MaxQuant enables high peptide identification rates, individualized p.p.b.-range mass accuracies and proteome-wide protein quantification. Nat. Biotechnol..

[41] Schaab C., Geiger T., Stoehr G., Cox J., Mann M. (2012). Analysis of High Accuracy, Quantitative Proteomics Data in the MaxQB Database. Mol. Cell. Proteom..

[42] Kong A.T., Leprevost F.V., Avtonomov D.M., Mellacheruvu D., Nesvizhskii A.I. (2017). MSFragger: Ultrafast and comprehensive peptide identification in mass spectrometry–based proteomics. Nat. Methods.

[43] Li H., Hou X., Du T. (2023). Responses of tomato fruit water balance and xylem hydraulic property of pedicel and calyx to water deficit and salinity stress. Environ. Exp. Bot..

[44] Braun D.M. (2022). Phloem Loading and Unloading of Sucrose: What a Long, Strange Trip from Source to Sink. Annu. Rev. Plant Biol..

[45] Sun C., Wei J., Gu X., Wu M., Li M., Liu Y., An N., Wu K., Wu S., Wu J. (2024). Different multicellular trichome types coordinate herbivore mechanosensing and defense in tomato. Plant Cell.

[46] Kim J.Y., Symeonidi E., Pang T.Y., Denyer T., Weidauer D., Bezrutczyk M., Miras M., Zöllner N., Hartwig T., Wudick M.M. (2021). Distinct identities of leaf phloem cells revealed by single cell transcriptomics. Plant Cell.

[47] Kuntorini E.M., Sari S.G., Fariani R. (2023). The morphoanatomy, histochemistry, and phytochemistry of the leaves and fruits of Rhodomyrtus tomentosa. Biodivers. J. Biol. Divers..

[48] Beckman C.H. (2000). Phenolic-storing cells: Keys to programmed cell death and periderm formation in wilt disease resistance and in general defence responses in plants?. Physiol. Mol. Plant Pathol..

[49] Wright B.W., Yi Z., Weissman J.S., Chen J. (2022). The dark proteome: Translation from noncanonical open reading frames. Trends Cell Biol..

[50] Kulkarni P., Uversky V.N. (2018). Intrinsically Disordered Proteins: The Dark Horse of the Dark Proteome. Proteomics.

[51] Medvedev K.E., Pei J., Grishin N.V. (2022). DisEnrich: Database of enriched regions in human dark proteome. Bioinformatics.

[52] Liu Y., Wu J., Sun N., Tu C., Shi X., Cheng H., Liu S., Li S., Wang Y., Zheng Y. (2017). Intrinsically Disordered Proteins as Important Players during Desiccation Stress of Soybean Radicles. J. Proteome Res..

[53] Das Laha S., Das D., Ghosh T., Podder S. (2023). Enrichment of intrinsically disordered residues in ohnologs facilitates abiotic stress resilience in Brassica rapa. J. Plant Res..

[54] Liu J., Liu J., Deng L., Liu H., Liu H., Zhao W., Zhao Y., Sun X., Fan S., Wang H. (2023). An intrinsically disordered region-containing protein mitigates the drought–growth trade-off to boost yields. Plant Physiol..

[55] Cuevas-Velazquez C.L., Vellosillo T., Guadalupe K., Schmidt H.B., Yu F., Moses D., Brophy J.A., Cosio-Acosta D., Das A., Wang L. (2021). Intrinsically disordered protein biosensor tracks the physical-chemical effects of osmotic stress on cells. Nat. Commun..

[56] Zhang M., Zhu C., Duan Y., Liu T., Liu H., Su C., Lu Y. (2022). The intrinsically disordered region from PP2C phosphatases functions as a conserved CO_2_ sensor. Nat. Cell Biol..

[57] Orand T., Delaforge E., Lee A., Kragelj J., Tengo M., Tengo L., Blackledge M., Boeri Erba E., Davis R.J., Palencia A. (2025). Bipartite binding of the intrinsically disordered scaffold protein JIP1 to the kinase JNK1. Proc. Natl. Acad. Sci. USA.

[58] Hansen J.C., Lu X., Ross E.D., Woody R.W. (2006). Intrinsic Protein Disorder, Amino Acid Composition, and Histone Terminal Domains. J. Biol. Chem..

[59] Wetzler D.E., Fuchs Wightman F., Bucci H.A., Rinaldi J., Caramelo J.J., Iusem N.D., Ricardi M.M. (2018). Conformational plasticity of the intrinsically disordered protein ASR1 modulates its function as a drought stress-responsive gene. PLoS ONE.

[60] Xoconostle-Cázares B., Martínez-Navarro A.C., Ruiz-Medrano R. (2016). Phloem Long-Distance Trafficking of RNAs and Proteins. eLS.

[61] Kehr J., Kragler F. (2018). Long distance RNA movement. New Phytol..

[62] Tolstyko E.A., Lezzhov A.A., Morozov S.Y., Solovyev A.G. (2020). Phloem transport of structured RNAs: A widening repertoire of trafficking signals and protein factors. Plant Sci..

