# Supplementary material for: The Analysis of Solanum lycopersicum Sap Dark Proteome Reveals Ordered and Disordered Protein Abundance
Source: Curr Issues Mol Biol. 2025 Sep 18;47(9):769. doi: 10.3390/cimb47090769 (PMC12468555; doi:10.3390/cimb47090769)
Supplement: Supplementary file 1 [file cimb-47-00769-s001.zip › supp_table_1.pdf]

Supplementary Table S1. Reanalyzed datasets.

Experiment number	Dataset identifier	Proteomic data acquisition	Biological sample	Condition	Year	Reference
1	PXD027940	Shotgun proteomic approach (GELC-MS)	Fruits	Mutant plants	2022	https://doi.org/10.1111/tpj.15925
2	PXD021973	Label free LC-MS/MS shotgun	Xylem sap	Excess Manganese	2020	https://doi.org/10.3390/ijms21228863
3	PXD018993	Label free LC-MS/MS shotgun	Phloem exudate	Drought and recovery	2020	https://doi.org/10.3390/ijms21124461
4	PXD008283	Label free quantitative GelC-LTQ-Orbitrap MS	Pollen grain	Heat Stress	2018	https://doi.org/10.3389/fpls.2018.01558
5	PXD010806	Label free quantitative nanoLC-MS/MS	Fruit peel	Healthy, Comparison between tissues	2018	https://doi.org/10.1021/acs.analchem.8b03005
6	PXD008762	Label free quantitative GelC-LTQ-Orbitrap MS	Pollen grain	Heat Stress	2018	https://doi.org/10.1186/s12864-018-4824-5
7	PXD007517	Label free LC-MS/MS shotgun	Xylem sap	Fe and Mn deficiencies	2018	https://doi.org/10.1016/j.jprot.2017.08.018
8	PXD003154	Label free LC-MS/MS shotgun	Trichomes	Healthy, Comparison between cultivars	2017	https://doi.org/10.1105/tpc.17.00060
