# Supplementary material for: The Analysis of Solanum lycopersicum Sap Dark Proteome Reveals Ordered and Disordered Protein Abundance
Source: Curr Issues Mol Biol. 2025 Sep 18;47(9):769. doi: 10.3390/cimb47090769 (PMC12468555; doi:10.3390/cimb47090769)
Supplement: Supplementary file 1 [file cimb-47-00769-s001.zip › supp_table_2.pdf]

Supplementary Table S2. UPs distribution within sets.

Names	total	elements
2_xylem 3.phloem_ibaq 8.trichomas_leaves	1	Solyc11g071260.2.1
3.phloem_ibaq 5.peridermis 8.trichomas_leaves	1	Solyc02g071320.4.1
2_xylem 6.pollen_calor	1	Solyc06g051100.1.1
2_xylem 7.xylem_soap_fe_mn	1	Solyc03g007930.4.1
3.phloem_ibaq 8.trichomas_leaves	8	Solyc11g068515.1.1 Solyc04g074770.2.1<br>Solyc07g049630.1.1 Solyc04g049930.2.1<br>Solyc04g015820.1.1 Solyc09g082810.3.1<br>Solyc09g075880.3.1 Solyc05g008140.4.1
6.pollen_calor 8.trichomas_leaves	1	Solyc11g066460.1.1
2_xylem	4	Solyc12g013520.3.1 Solyc12g035380.1.1<br>Solyc06g009450.1.1 Solyc01g020521.1.1
3.phloem_ibaq	8	Solyc07g064860.2.1 Solyc11g012325.1.1<br>Solyc05g005500.1.1 Solyc04g082915.1.1<br>Solyc03g044420.2.1 Solyc04g078450.4.1<br>Solyc12g062680.3.1 Solyc08g082190.3.1
5.peridermis	2	Solyc08g065627.1.1 Solyc02g005330.1.1
6.pollen_calor	14	Solyc12g006400.2.1 Solyc04g024820.1.1<br>Solyc12g056320.1.1 Solyc04g011615.1.1<br>Solyc04g051720.3.1 Solyc07g043280.1.1<br>Solyc12g038660.1.1 Solyc09g009930.2.1<br>Solyc11g072670.1.1 Solyc03g114700.2.1<br>Solyc03g051665.1.1 Solyc00g500174.1.1<br>Solyc00g500159.1.1 Solyc00g500266.1.1
7.xylem_soap_fe_mn	2	Solyc06g030660.3.1 Solyc01g004006.1.1
8.trichomas_leaves	17	Solyc01g019100.1.1 Solyc08g082130.3.1<br>Solyc11g073231.1.1 Solyc02g150127.1.1<br>Solyc04g080310.1.1 Solyc04g026040.1.1<br>Solyc08g077825.1.1 Solyc03g112220.1.1<br>Solyc03g123710.3.1 Solyc02g077980.3.1<br>Solyc07g052310.1.1 Solyc09g082920.1.1<br>Solyc09g150129.1.1 Solyc02g087975.1.1<br>Solyc02g082020.3.1 Solyc10g081000.1.1<br>Solyc06g082350.4.1
